# Supplementary material for: Learning curve and surgical outcome of robotic assisted colorectal surgery with ERAS program
Source: Sci Rep. 2022 Nov 29;12:20566. doi: 10.1038/s41598-022-24665-w (PMC9709162; doi:10.1038/s41598-022-24665-w)
Supplement: Supplementary file 1 — Supplementary Information 1. [file 41598_2022_24665_MOESM1_ESM.zip › LSC Raw data-Ñ[▒K/061/O_ñw╝╨░O▒Kñσ.pdf]

正式摘要(電子簽章：00000184299685)

請選擇...reporter

臺中榮民總醫院

Operation Note

[redacted]

- 手術日期：2020/01/10
- 手術醫師：VS 蔣鋒帆，CR 江世偉，R 林陳彥，R 邱俊凱
- Anesthesia：GE
- Position：Lithotomy
- Aseptic agents：Betadine Alcohol，Betadine Aqua

Diagnosis

- Pre-operative diagnosis : Malignant tumor of rectum,DENTATE LINE
- Post-operative diagnosis : Ditto.

Surgical Indication

- For tumor resection

Operation

- 1. Laparoscopic low anterior resection, colo-anal anastomosis, pull through method.
  2. Ileostomy at RLQ
  3. High ligation (-) below left colic artery
  4. Cut margin: proximal 13 cm, distal 0.5 cm
  5. Take down of splenic flexure: (-)
  6. A 12mm penrose drain placed in the pelvis

Finding

- 1. A 2.0*1.5cm Bormann's type III tumor at lower rectum, just at dentate line, invaded to the muscular layer but didn't penetrate through the serosa. No enlarged lymph nodes at mesorectum, ycT2NM0 (s/p short course radiotherapy)
  2. No synchronous tumor nor polyps at resected colon.
  3. There was no palpable liver metastasis, no visible peritoneal seeding

Procedure

- General endotracheal anesthesia was induced and then the patient was placed in modified Trendelenburg lithotomy position. Preoperative antibiotics were given. A Foley catheter and nasogastric tube were placed. The abdomen was prepped and draped in standard sterile fashion. A small vertical incision was made at low midline and RLQ and 12mm trochars were inserted into the peritoneal cavity by open method. Peumoperitoneum was established with CO2 inflation. Laparoscopy was inserted and then another two 5mm trochars were inserted over LLQ and lower midline of abdomen. The operative findings were mentioned as above. The small bowel was inspected and retracted to the right by grasper. The sigmoid colon was then held by grasper and the peritoneum overlying the mesentery was then scored with electrocautery. The inferior mesentery artery was identified, ligated with endoclips, and transected, so was inferior mesentery vein. Dissection was done above the Gerota's fascia and toward the peritoneal attachments. The colon was mobilized from retroperitoneum overlying the sacral promontory and into the pelvis. The arcade vessels to the sigmoid colon were also divided. Distal descending colon and rectum were mobilized to the level of levator ani. Attention was then turned to the perineum. The anus was carefully dilated and well exposed with stay sutures. The pelvis was entered after incision through anus. The rectum and sigmoid was then delivered through anus without tension or torsion. Coloanal anastomosis was constructed using mayo scissors and whole layer of interrupted 3-0 vicryl sutures. One 12mm penrose drain was then placed at pelvis. A transverse incision was made at the RLQ of abdomen, right at trochar incision. The terminal ileum was pulled out of the abdomen. A window was created

- under the ileum and a plastic bridge was placed at this point. The fascia was closed with interrupted sutures of No.1 Vicryl. The skin was closed with 3-0 Nylon. The ileostomy was then mature with multiple interrupted sutures of 3-0 monocryl placed in such a way as to tack the full thickness of the edge of the transverse colon to a subcuticular layer of the skin. An ostomy appliance was placed. The patient tolerated the procedure well and was brought to the POR in stable condition.
- Estimated blood loss: minimum
- 器械數量: 正確
- 紗布數量: 正確

Image

- PAINT
  - abdomen

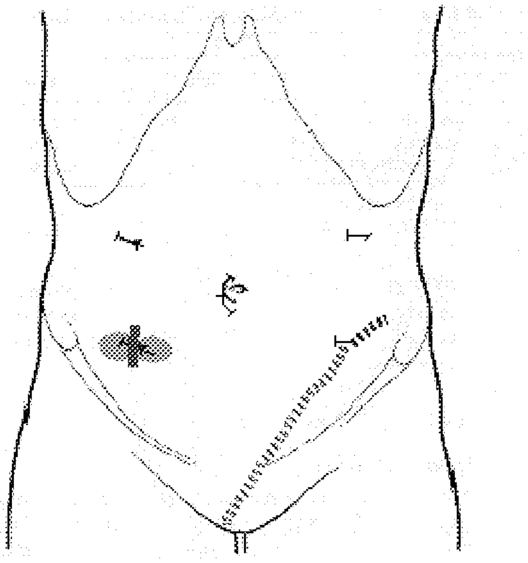

  - colon & rectum

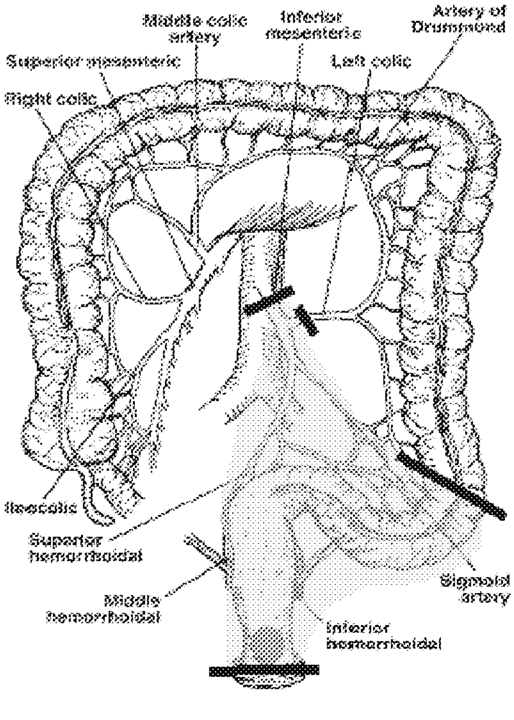

編輯主任 2062F蔣鋒帆、主治 2288K江世偉

正式摘要(電子簽章：000000184299685)

請選擇...reporter

doctorRank:B
